# Systematic analysis of bacteriostatic mechanism of flavonoids using transcriptome and its therapeutic effect on vaginitis

**DOI:** 10.18632/aging.103024

**Published:** 2020-04-09

**Authors:** Zeyan Lin, Yanyan Lin, Zhengbing Zhang, Jinxing Shen, Caimei Yang, Meijiao Jiang, Yongming Hou

**Affiliations:** 1 State Key Laboratory of Ecological Pest Control for Fujian and Taiwan Crops, Fujian Agriculture and Forestry University, Fuzhou, China; 2 Fujian Provincial Key Laboratory of Insect Ecology, Department of Plant Protection, Fujian Agriculture and Forestry University, Fuzhou, Fujian, China; 3 Department of Pharmacy, Zhangzhou Health Vocational College, Zhangzhou, Fujian, China; 4 Faculty of Basic Medicine, Zhangzhou Health Vocational College, Zhangzhou, Fujian, China; 5 Biobank, The First Affiliated Hospital of Xiamen University, Xiamen, Fujian Province, China; 6 Zhangzhou Affiliated Hospital of Fujian Medical University, Zhangzhou, Fujian, China

**Keywords:** flavonoids, antibacterial effect, vaginitis, transcriptome

## Abstract

The flavonoids in *Ageratum conyzoides* L. have been used in traditional medicine due to its anti-inflammatory and antibacterial properties. However, the specific mechanism of its antibacterial effect, and the potential therapeutic effect on vaginitis have not been well explained. The growth curves of E. coli, S. aurues, and P. aeruginosa after treatment with flavonoids were measured. The influences of flavonoids on the conductivity of bacterial culture medium and exudation of bacterial nucleic acid were also detected. Transcriptomics analysis was applied to analyze the potential mechanism of flavonoids. Flavonoids significantly suppressed the growth curves of E. coli, S. aurues, and P. aeruginosa, and increased the conductivity of bacteria and nucleic acid exudation. Transcriptomics analysis indicated that flavonoids could suppress bacteria by affecting the transcription and metabolism pathways. The obvious therapeutic effect of flavonoids on bacterial vaginitis was also observed. This study systematically analyzed the bacteriostatic mechanism of flavonoids, which should be helpful to develop new drugs based on the bacteriostatic effect of flavonoids.

## INTRODUCTION

Nowadays, antimicrobial resistance (AMR) is an ineluctable biological process due to the improper use of antibiotics in humans, animals and the environment, which can threaten human health as well as agricultural livelihoods and global food security1. In 2016, the World Banks’ report estimated that the global gross domestic product in low income countries will fall by exceeding 5% by 2050, if we do not take action to interfere with the AMR progress [1]. AMR is not only a concern for low and middle income countries, but also results in at least two million resistant infections and at least 23 000 deaths in the United States each year [2]. Hence, AMR is considered a global public health and economic problem [3], and there is an urgent need to develop more new suitable alternative antimicrobial agents for the treatment of infectious diseases.

Flavonoids are a class of natural compounds that are ubiquitous in photosynthesising cells and are generally found in fruit, vegetables, nuts, seeds, stems, flowers, tea, wine, propolis and honey [4]. Flavonoids possess a variety of biological functions, such as antioxidation, anti-hyperlipidemia, anti-fatigue, anti-aging, and atherosclerosis-prevention activities [5]. Flavonoids are also well known for their antimicrobial capabilities. For example, it has been reported that glabrol, a flavonoid from licorice, displayed high efficiency against methicillin-resistant Staphylococcus aureus (MRSA) in both *in vivo* and *in vitro* models [6]. A new class of modified flavonoids namely apigenin and quercetin was found to completely inhibit the growth of Listeria monocytogenes, Pseudomonas aeruginosa, and Aeromonas hydrophila [7]. Some synthetic derivatives of flavonoids also displayed 20- to 80-fold more potent antimicrobial capacity than standard antibiotics against multidrug-resistant Gram-negative and Gram-positive bacteria [8].

*Ageratum conyzoides* L. (*A. conyzoides*) is an annual herb that is commonly used as a traditional medicine in many countries around the world, especially in the tropical and subtropical regions [9]. To date, the antimicrobial capacity of *A. conyzoides* has been reported by several studies [10, 11]. Flavonoids can be extracted from *A. conyzoides* [9], and their roles in antibacterial activities have not been well elucidated. In the present study, we aimed to detect the effects of flavonoids in *A. conyzoides* on Gram-negative and Gram-positive bacteria, and to explore the underlying mechanisms. Furthermore, the impact of flavonoids in *A. conyzoides* on the bacterial vaginitis in mice was also tested.

## RESULTS

### Antibacterial effects of flavonoids purified from A. conyzoides

**Figure 1:**
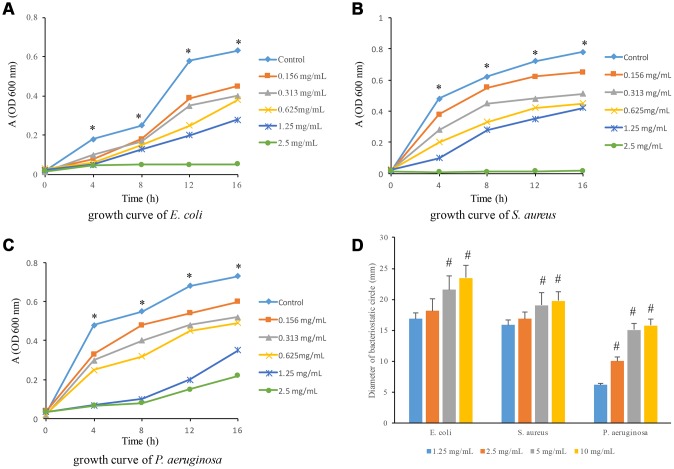
**Antibacterial effects of flavonoids in *A. conyzoides on* S. aureus, E. coli and P. aeruginosa.** (**A**–**C**) The growth curves of S. aureus, E. coli and P. aeruginosa upon the administrations of gradient doses of flavonoids in *A. conyzoides*, respectively. The horizontal coordinate represents the reaction time, and the vertical coordinate represents the absorption value at 600 nm. (**D**) Flavonoids in *A. conyzoides* significantly enlarged the bacteriostatic circles of S. aureus, E. coli and P. aeruginosa in a concentration-dependent manner. * P< 0.05 compared with treated groups; #<0.05 compared with group 1.25 mg/mL.

The suppressive effects of flavonoids on E. coli, S. aurues, and P. aeruginosa were measured. After treatment with 2.5 mg/mL flavonoids, the growth curve of S. aureus and E. coli is flat without significant rising trend, suggesting that the minimum antibacterial concentration of flavonoids to S. aureus and E. coli was 2.5 mg/mL (Figure 1A and 1B). However, 2.5 mg/mL flavonoids did not show sustained antibacterial effects on P. aeruginosa after 8 hours. Therefore, the minimum antibacterial concentration of P. aeruginosa could not be ascertained by this growth curve (Figure 1C). Moreover, the growth curves of all flavonoids treated groups were under the curves of control group, indicating that even the 0.156 mg/mL flavonoids presented significant antibacterial effects. We also detected the influences of flavonoids at different concentrations on the bacteria inhibition zones. With the increase of flavonoids concentration, the diameter of inhibition zones was significantly increased (Figure 1D).

### Influence of flavonoids on conductivity of bacterial culture medium and exudation of bacterial nucleic acid

**Figure 2:**
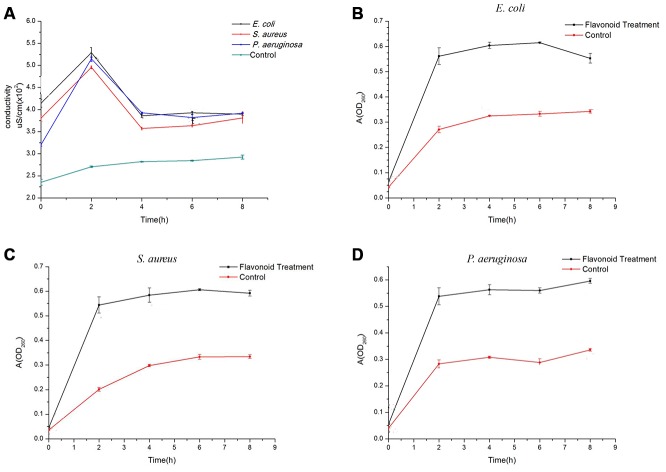
**Effects of flavonoids in *A. conyzoides* on the conductivity of bacterial culture medium and the nucleic acid exudation in S. aureus, E. coli and P. aeruginosa.** (**A**) Compared to the control group, flavonoids in *A. conyzoides* significantly elevated the culture medium conductivities of S. aureus, E. coli and P. aeruginosa to the peak after administrated for 2 hours. Such conductivities dropped to a relative steady status since the 4^th^ hour. (**B**–**D**) The absorption value at 260 nm was recorded, which represents the nucleic acid exudation in bacteria. Upon the administration of flavonoids in *A. conyzoides* for 2 hours, the nucleic acid exudation in S. aureus, E. coli and P. aeruginosa was remarkably raised to the peak, followed by a relative steady status, respectively.

After incubation with flavonoids for 2 hours, the conductivity of E. coli (5.290 ± 0.115 μS/cm), S. aurues (4.957 ± 0.032 μS/cm), and P. aeruginosa (5.150 ± 0.058 μS/cm) increased remarkably compared to the control group (2.707 ± 0.015μS/cm), suggesting that the permeability of cell membrane is the largest at this time (Figure 2A). After treatment for 4 hours, the conductivity of three kinds of bacteria decreased in a stable level, but was still significantly higher than the control group. In addition, based on the results of conductivity curve, it can be concluded that the best reaction time of flavonoids on three kinds of bacteria is 0-4 h, which is consistent with the clinical medication guidance.

The ultraviolet absorption of three kinds of bacteria increased sharply after treatment for 2 hours compared to the control group. While, after incubation for 4 hours, the absorbance of three treatment groups tended to be stable, but still was remarkably higher than that of the control group (Figure 2B–2D). These changes were consistent with the findings of conductivity experiments. It can be determined that with the increase of the treatment time, the permeability of bacterial membrane increased, and the DNA and RNA in the bacteria were released into the solution, which improved the absorption value at 260 nm.

### Transcriptomics analysis of *E. coli* and *S. aureus* after treatment with flavonoids

**Figure 3:**
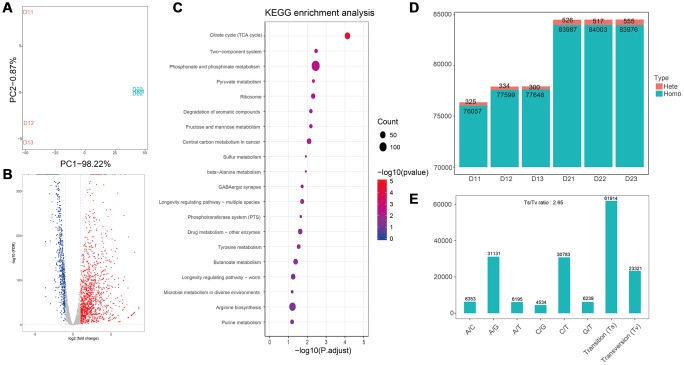
**Transcriptome analyses of *E. coli* after treatment with flavonoids in *A. conyzoides*.** (**A**) PCA plot for *E. coli* with or without the treatment of flavonoids in A.conyzoides. (**B**) Volcano Plot of expression of genes in E. coli after treatment with flavonoids in *A. conyzoides*. The horizontal coordinate represents the log2 value taking from the fold changes of gene expression, and the vertical coordinate represents the p-value with -log10 transformed. The red, blue and gray dots represent up-regulation, down-regulation and none difference, respectively. (**C**) KEGG enrichment analysis of E. coli after treatment with flavonoids in *A. conyzoides* with top 20 pathways presented. The horizontal coordinate represents the Rich Factor, and the vertical coordinate represents the KEGG Pathway entry. The size of the dot in the graph indicates the number of differential genes annotated to the pathways, and the color shade represents the significant FDR value of the pathways. The graph only displays the twenty most significantly altered pathways. (**D**, **E**) Quantity statistics of the SNPs and the Transition/Transversion of genotype in E. coli after treatment with flavonoids in *A. conyzoides*.

**Figure 4:**
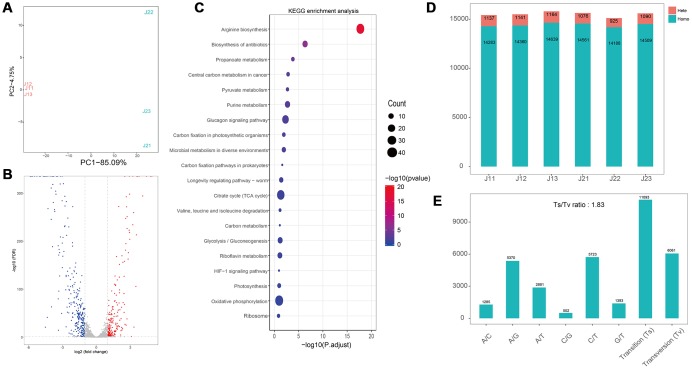
**Transcriptome analyses of S. aureus after treatment with flavonoids in *A. conyzoides*.** (**A**) PCA plot for *S. aureus* with or without the treatment of flavonoids in A.conyzoides. (**B**) Volcano Plot of Unigene expression changes in S. aureus after treatment with flavonoids in *A. conyzoides*. The horizontal coordinate represents the log2 value taking from the fold changes of gene expression, and the vertical coordinate represents the p-value with -log10 transformed. The red, blue and gray dots represent up-regulation, down-regulation and none difference, respectively. (**C**) KEGG enrichment analysis of S. aureus after treatment with flavonoids in *A. conyzoides* with top 20 pathways presented. The horizontal coordinate represents the Rich Factor, and the vertical coordinate represents the KEGG Pathway entry. The size of the dot in the graph indicates the number of differential genes annotated to the pathways, and the color shade represents the significant FDR value of the pathways. The graph only displays the twenty most significantly altered pathways. (**D**, **E**) Quantity statistics of the SNPs and the Transition/Transversion of genotype in S. aureus after treatment with flavonoids in *A. conyzoides*.

PCA analysis suggested the good biological duplication in our data, and the apparent difference between control and treatment samples (Figures 3A, and 4A). Differentially expression genes (DEG) were identified with edgeR (version 3.28.0). After treated with flavonoids, a total of 669 genes were down-regulated in E.coli samples, with 1161 genes were up-regulated (Figure 3B). Based on the differentially expressed genes, KEGG pathways were analyzed, and the main metabolic pathways and signal transduction pathways involved in the differentially expressed genes could be obtained. The top 20 enriched pathways were shown in Figure 3C. Among the 20 enriched pathways, purine metabolism has the most significant difference, 15 pathways were related to metabolism, 3 pathways were related to organizational systems, and 2 pathways were related to environmental information processing and genetic information processing, respectively (Figure 3C). The SNP analyses of control samples (D11, D12, and D13) and flavonoids treated samples (D21, D22, and D23) were conducted. Remarkable difference in the number of total cSNP between the control and flavonoids treated group was observed (Figure 3D). We further analyzed the transition and transversion of SNP base types, and found that the transition ratio of A/G and C/T was higher, the transition ratio of C/G was lower, and the number of base transition (61914) was much higher than that of base transversion (23321) (Figure 3E).

Moreover, the flavonoids treated S. aureus was also analyzed with transcriptomics method. After treatment with flavonoids, the number of up-regulated genes was 202, and the number of down-regulated genes was 289 (Figure 3B). Compared to E. coli group, low proportion of differential genes was observed in the S. aureus group. The top 20 enriched pathways were presented in Figure 4C, indicating that 16 pathways were correlated with metabolism, 2 pathways were linked with genetic information processing and 2 pathways were related to environmental information processing and organismal systems, respectively (Figure 4C). We found that there was no significant difference in the number of homozygous cSNP, heterozygous cSNP and total cSNP between different groups (Figure 4D). Analyses of transition and transversion of SNP base types indicated that the transition ratio of A/G and C/T was higher, and the number of base transition (11093) was much higher than that of base transversion (6061) (Figure 4E).

### Influence of flavonoids on biochemical indexes of liver and kidney metabolism and inflammatory cytokines

**Figure 5:**
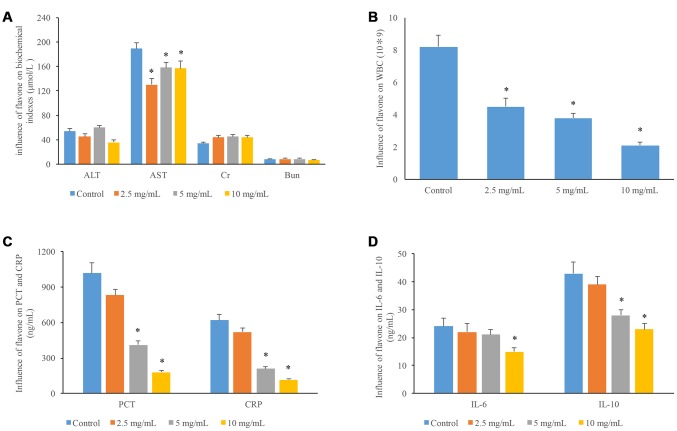
**Influences of flavonoids in *A. conyzoides* on liver and kidney function, and the biochemical indexes of inflammation in mice.** (**A**) Compared to the control group, various doses of flavonoids in *A. conyzoides* did not significantly affect the serum levels of ALT (a biomarker of hepatic function), Cr and Bun (biomarkers of kidney function) in mice. While the serum level of AST (another biomarker of hepatic function) was significantly decreased by various doses of flavonoids in *A. conyzoides* (*P<0.05, compared to control). (**B**) The number of white blood cell (WBC) was reduced by flavonoids in *A. conyzoides* in a dose-dependent manner (*P<0.05, compared to control). (**C**) Flavonoids in *A. conyzoides* at 5 and 10 mg/mL significantly decreased the serum levels of two biochemical indexes of inflammation namely procalcitonin (PCT) and C-reactive protein (CRP) in mice (*P<0.05, compared to control). (**D**) Flavonoids in *A. conyzoides* at 5 or 10 mg/mL significantly reduced the serum levels of two pro-inflammation cytokines namely IL-6 and IL-10 in mice (*P<0.05, compared to control).

Three different concentrations of flavonoids were used to treat mice, and several physiological items were measured. The high concentration of flavonoids (10 mg/mL) obviously reduced the ALT levels in mice, and all the three concentrations of flavonoids significantly decreased the AST levels in mice compared to group control. Meanwhile, the CR and BUN levels in mice were increased after treatment with flavonoids, but the increases were not significant (Figure 5A). These findings indicate that flavonoids have no significant effects on the metabolism of liver and kidney in mice. In addition, the levels of WBC, PCT, and CRP were markedly suppressed by the treatment with middle and high concentrations of flavonoids (Figure 5B and 5C). Moreover, significant decrease in IL-6 and IL-10 levels was observed after treatment with 10 mg/ml flavonoids (Figure 5D).

### Therapeutic effects of flavonoids on bacterial vaginitis

**Figure 6:**
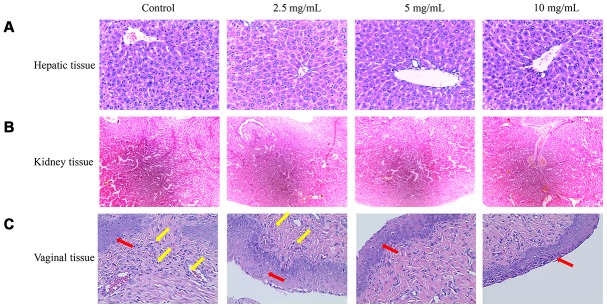
**Therapeutic effects of flavonoids in *A. conyzoides* on bacterial vaginitis in mice.** (**A**, **B**) HE staining showed that various doses of flavonoids in *A. conyzoides* did not induce obvious histomorphologic changes in liver and kidney tissues of mice. (**C**) In the control group, the squamous cells in the basal layer of vagina tissue were damaged and a large number of inflammatory cells were infiltrated in vagina tissue, indicating that the bacterial vaginitis model in mice was successfully established. After treatment with low concentration of flavonoids in *A. conyzoides* (2.5 mg/mL), fewer inflammatory cells were observed. Furthermore, after treatment with middle and high concentrations of flavonoids in *A. conyzoides* (5 mg/mL and 10 mg/mL), no obvious inflammatory cell infiltration in vagina tissue was observed and the squamous cells in the basal layer were arranged regularly and smoothly, indicating that inflammation in vagina tissue was remarkably improved. Red arrows point to squamous cells and yellow arrows point to inflammatory cells.

Three concentrations of flavonoids possessed no significant damage on the mice liver. After treatment with low concentration of flavonoids (2.5 mg/mL), a few cells were lack of nucleus, but most of the cells grew well with a small amount of mitosis. After treatment with middle and high concentrations of flavonoids (5 mg/mL and 10 mg/mL), the hepatocytes also grew well without inflammatory cell infiltration and with less congestion in the hepatic sinuses (Figure 6A). No significant pathological changes were found in the renal tissue sections of each treatment group. Smooth glomerulus, normal medulla and cortex were observed, and these results were consistent with the conclusion of biochemical items (Figure 6B).

In the control group, the squamous cells in the basal layer of the vagina were destroyed, a large number of nuclei were lost in the cervical columnar cells, and a large number of inflammatory cells were infiltrated in the tissues, indicating that the vaginitis model was successfully established (Figure 6C). After treatment with low concentration of flavonoids (2.5 mg/mL), few inflammatory cells were observed. Moreover, after treatment with middle and high concentrations of flavonoids (5 mg/mL and 10 mg/mL), no infiltration of inflammatory cells in the tissue were observed, the squamous cells in the basal layer were arranged regularly and smoothly, and no hyperplasia was found, indicating that there was no chronic inflammation (Figure 6C).

## DISCUSSION

In the present study, we demonstrated that total flavonoids in *A. conyzoides* could significantly inhibit the growth of S. aureus, E. coli and P. aeruginosa in a concentration-dependent manner, and could significantly increase the membrane permeability, which could lead to nucleic acid exudation in S. aureus, E. coli and P. aeruginosa. Moreover, based on the transcriptomics analysis, we detected that transcription levels of many signal pathways in S. aureus and E. coli were significantly altered upon the administration of the flavonoids in *A. conyzoides*. Finally, we found that the flavonoids in *A. conyzoides* could prominently improve the bacterial vaginitis in mice without obvious liver and kidney damages.

To our knowledge, the antiprotozoal activity of flavonoids in *A. conyzoides* has been previously reported [12, 13], while their antibacterial activities have not been well clarified. In the current study, we. revealed for the first time that flavonoids in *A conyzoides* possessed significant antibacterial effects on S. aureus, E. coli and P. aeruginosa. Moreover, we found that flavonoids in *A. conyzoides* significantly elevated the medium conductivities of S. aureus, E. coli and P. aeruginosa after administrated for 2 hours, indicating that the membrane permeability of all the three kinds of bacteria was significantly increased. In addition, we detected that the DNA and RNA exudations of S. aureus, E. coli and P. aeruginosa were also remarkably enhanced after the administration of flavonoids in *A. conyzoides* for 2 hours. There have been several evidences showed that flavonoids could exert their antibacterial actions via inducing membrane permeabilization [14–16]. Hence, we speculate that the antibacterial mechanism of flavonoids in *A. conyzoides* is that they can also cause membrane permeabilization and consequent nucleic acid exudation in bacteria.

Subsequently, we detected that the flavonoids in *A. conyzoides* significantly decreased the gene expression levels of metabolism pathways in E. coli, especially the nucleotide metabolism pathway, the energy metabolism pathway and the carbohydrate metabolism pathway using the KEGG enrichment analysis. These results were consistent with previous reports that the flavonoids could exert their anti-E. coli effects by inhibiting DNA gyrase and ATP synthase [17]. Furthermore, we found that the number of SNPs in flavonoids treated E. coli was significantly increased, indicating that the flavonoids in *A. conyzoides* could significantly enhance the probability of nucleotides mutation in E. coli, which is speculated to be an important reason for the significant changes in the transcription levels of E. coli. Whereas, we detected that major pathways influenced by flavonoids in *A. conyzoides* in S. aureus were the translation and carbon metabolism pathways, and the number of SNPs was not significantly altered by the flavonoids in *A. conyzoides*. These data suggest that there is a certain difference in the mechanism of antimicrobial effects of the flavonoids in *A. conyzoides* on E. coli and S. aureus, which may be ascribed to the differences between their own characteristics. Moreover, these results indicate that the flavonoids in *A. conyzoides* may possess more prominent antimicrobial effects on E. coli than S. aureus, which is consistent with the data shown in Figure 1D that the bacteriostatic circle of flavonoids treated E. coli was significantly bigger than that of flavonoids treated S. aureus.

In recent years, plenty of studies have revealed the anti-inflammation activities of flavonoids. For instant, it has been reported that pillion, a flavonoid compound from Aquilaria sinensis, could significantly reduce the serum levels of pro-inflammatory cytokines namely IL-6 and TNF-α in LPS-induced septic mice [18]. Furthermore, pinocembrin that is one of the primary flavonoids in propolis has been proved to significantly attenuate allergic airway inflammation via inhibition of NF-κB pathway in mice [19]. In the present study, we also observed that flavonoids in *A. conyzoides* exhibited obvious anti-inflammation effects on bacterial vaginitis in mice. To our knowledge, *A. conyzoides* is widely used as a traditional medicine to cure various diseases due to its anti-inflammatory, antinociceptive and antibacterial properties [20]. However, the safety of phytoconstituents in *A. conyzoides* is still uncertain at present. It has been reported that alkaloids extracted from *A. conyzoides* were found responsible for inducing liver toxicity [21]. Moreover, a previous research conducted by Diallo et al. showed that hydroalcholic extract of *A. conyzoides* at 500 and 1000 mg/kg body weight could induce liver, kidney and haematological disorders [22]. In contrary, Antai et al. have reported that chronic treatment with the ethanolic leaf extract of *A. conyzoides* at 200, 400, and 600 mg/kg body weight did not significantly alter the expression levels of ALT and AST in serum and liver of all tested rats [23]. In our study, we also detected that flavonoids in *A. conyzoides* exhibited no obvious damages on the liver and kidney in experimental mice. Notably, we found that flavonoids in *A. conyzoides* significantly decreased the serum levels of AST in mice, indicating that flavonoids in *A. conyzoides* have protective effects on hepatic function in mice. This is consistent with results in many previous researches that flavonoids could exert considerable protections against the hepatic damages induced by various causes [24, 25].

Our findings in the current study suggest the potential of flavonoids in *A. conyzoides* as a source of new and safe medicines to treat infective diseases. However, a limitation of our study is that the data revealed by transcriptomics analysis should be further verified *in vivo* and *in vitro*. Furthermore, we did not elucidate the underlying molecular mechanism in protective activities of flavonoids in *A. conyzoides* against bacterial vaginitis, which should also be explored in future.

In summary, we demonstrated the antibacterial effects of flavonoids on E. coli, S. aurues, and P. aeruginosa. The conductivity of bacteria and nucleic acid exudation were increased significantly by flavonoids, which should be the potential antibacterial mechanism. Meanwhile, transcriptomics analysis of E. coli and S. aureus after treatment with flavonoids was conducted. The therapeutic effect of flavonoids on bacterial vaginitis was also validated.

## MATERIALS AND METHODS

### Materials

The flavonoids in *A. conyzoides* power, Staphylococcus aureus (S. aureus), Escherichia coli (E. coli), and Pseudomonas aeruginosa (P. aeruginosa) used in this study were purchased from herbal garden of Zhangzhou health vocational college (Fujian, China). The ICR female mice, purchased from the Animal Experimental Center of Fujian Medical University (certificate number: SCXK-Min-2016-0002). For quality control, the test of endotoxin content in the flavonoids power was measured using tachypleus amebocyte lysate, and the related results were presented in the Supplementary Materials (Supplementary Tables 1, 2).

### Preparation of bacterial suspension

The bacteria were cultured in broth medium (37 °C, 1200 rpm/min). After 24 hours, the bacterial suspension was collected and centrifuged in freezing centrifuge at 3500 rpm/min. Upper medium was discarded, and the bacteria were washed with PBS buffer for 3 times. The sterile normal saline was added gradually, and then the absorbance value was measured at 600 nm. When the absorbance value was 1, it represented 1 × 10^9^ cfu. Then the bacterial suspension was diluted according to the required concentration.

### Measurement of bacteriostatic circle

Flavonoids were diluted into 5 concentrations (10 mg/mL, 5 mg/mL, 2.5 mg/mL, 1.25 mg/mL, 0.625 mg/mL) with sterile ultra pure water. Bacteria suspension (1 mL, 107 cfu/mL) and sterilized liquid nutrient agar medium (15 mL, Life Technologies, California, China) were mixed. After solidification of medium, different concentrations of sterilized flavonoids were added. The bacteriostatic circle was examined 24 hours later.

### Measurement of bacterial growth curve after treatment with flavonoids

Different concentrations of flavonoids were prepared with broth medium in the 48-well plate, then 100 μL bacterial suspension was added to each well. The absorbance value at 600 nm was measured every 4 hours.

### Measurement of the conductivity of bacterial culture medium

The prepared bacterial suspension (2 mL, 10^7^ cfu) was added to test tube, and 2 mL flavonoids (2.5 mg/mL) were added. The suspension was cultured in constant temperature vibration incubator (120 rpm/min). 500 μL suspension was collected at 0 h, 2 h, 4 h, 6 h, and 8 h, and centrifuged at 5000 rpm/min for 10 min. After 20 times dilution, the supernatant was measured by conductivity meter (Leici, Shanghai, China).

### Measurement of the DNA and RNA exudation of bacteria

The prepared bacterial suspension (1 mL, 10^7^ cfu) and 100 μL flavonoids (2.5 mg/mL) were added to centrifuge tube. The suspension was collected at 0 h, 2 h, 4 h, 6 h, and 8 h, and centrifuged at 3500 rpm/min for 5 min. The absorbance value of supernatant at 260 nm was measured.

### Transcriptomics analysis of the bacteriostatic effect of flavonoids on E. coli, S. aurues

Bacterial suspension (10^7^ cfu, 100 ml) was prepared after centrifugation of E.coli in logarithmic growth period. 50 ml of each suspension was put into two 50 ml sterilized centrifuge tubes for standby. Flavonoids powder, 46.56% purity, was put into 50 mL bacterial suspension to prepare 0.625 mg/mL flavonoids suspension, which was recorded as D2. The other 50 mL bacterial suspension without treatment was recorded as D1. S. aurues was treated in the same way. The blank group was recorded as J1, and the flavonoids treatment group was marked as J2. D11, D12, D13, were the three repeated samples of D1, and D21, D22, D23 were the three repeated samples of D2. Likewise, J11, J12, J13 were the three repeated samples of J1, and J21, J22, J23 were the three repeated samples of J2. The Illumina hiseq second generation transcriptome sequencing was conducted by Shanghai paisenno Biotechnology Co., Ltd (Shanghai, China).

The original sequenced sequences (sequenced reads) obtained by the sequencer were filtered and spliced to obtain high-quality sequences, and the filtered sequences were compared to reference genomes downloaded from NCBI. Detailly, raw RNA-seq reads were processed with Trimmomatic [26] to remove low quality regions and adapter sequences. Clean reads were mapped to the reference genome of *E.coli* (https://www.ncbi.nlm.nih.gov/nuccore/218430358) or *S. aureus* (https://www.ncbi.nlm.nih.gov/assembly/GCF_000013425.1) using hisat2 [27]. Gene expression were summarized by HTseq-count [28]. Raw counts were further normalized to counts per million (CPM) and genes with CPM < 1 in 3 samples were regarded as lowly-expressed genes and were removed from further analysis. Differentially expressed genes were identified using edgeR [29] with significance thresholds of false discovery rate (FDR) < 0.05 and fold change > 2. Enrichment analyses with differentially expressed genes were carried out with clusterProfiler [30]. The transcriptional structure of the samples was analyzed; the operon substructure, UTR, cSNP, indel and other information of the sample genes were obtained.

### Animal experiment

The ICR female mice were treated with 0.1 mL mixed bacteria suspension (E. coli, S. aureus, P. aeruginosa, 1: 1: 1) daily through vaginal injection. After 5 days, obvious swelling and purulent flow out could be observed in the mouse vagina mouth. Then different groups of mice were treated daily with 0.1 mL different concentrations of flavonoids (0, 2.5, 5, and 10 mg/mL) through caudal vein injection for 7 days. Then the tissues and serum were collected for additional experiments. All experiments were approved by the Institutional Animal Care and Use Committee of Fujian Agriculture and Forestry University.

### HE staining

HE staining was performed as described previously [31, 32]. Briefly, liver, kidney, and vaginal tissues were separated after the sacrifice of mice. The tissues were fixed with 15% formalin for 24 h. OCT compound (Sigma, St. Louis, USA) was used for tissue embedding, and 12-μm thickness tissues were prepared with a frozen microtome. Three slides in each group were applied for HE staining. Zeiss AxioVision (Jena, Germany) was used for capturing images.

### Measurement of biochemical items in the serum

Blood was collected from the mouse retro-orbital sinus puncture under isoflurane anesthesia. After centrifugation (2000 r/min, 15 min), the serum was used for measuring the levels of procalcitonin (PCT), C-reactive protein (CRP), alanine aminotransferase (ALT), aspartate aminotransferase (AST), creatinine (Cr) and blood urea nitrogen (BUN) using an auto chemistry analyzer (Langpu, Beijing, China). The levels of interleukin-6 (IL-6) and interleukin-10 (IL-10) were measured with ELISA kits (Nanjing Jiancheng, Nanjing, China).

### Statistical analysis

Results are expressed as mean ± standard error (SEM). Statistical analysis was performed using one-way analysis of variance (ANOVA). P < 0.05 means statistically significant. Statistical analysis was performed using SPSS 19.0 (SPSS Co., Ltd., USA).

## Supplementary Material

Supplementary Information

Supplementary Tables
