# Supplementary material for: Systematic analysis of bacteriostatic mechanism of flavonoids using transcriptome and its therapeutic effect on vaginitis
Source: Aging (Albany NY). 2020 Apr 9;12(7):6292–305. doi: 10.18632/aging.103024 (PMC7185132; doi:10.18632/aging.103024)
Supplement: Supplementary Information [file aging-12-103024-s001..pdf]

## SUPPLEMENTARY INFORMATION

### Measurement of endotoxin content in the flavonoids

The tachypleus amebocyte lysate was purchased from Xiamen tachypleus amebocyte lysate factory (# 170718). The sensitivity test of tachypleus amebocyte lysate was firstly conducted. Briefly, standard of endotoxin (1 EU/mL) was diluted to 0.5 EU/mL, 0.25/EU·mL, 0.125 EU/mL, 0.062 EU/mL, and 0.031 EU/mL respectively. Different concentrations of endotoxin were added to different ampoule bottles. The tachypleus amebocyte lysate was resolved using detection water, added to different ampoule bottles. Then the solution was incubated in the endotoxin detector, and the gel generation was investigated. The experiment was conducted twice.

Based on the calculation formula ($\lambda c = lg\text{-}1(\Sigma x/4)$) of sensitivity test of tachypleus amebocyte lysate, c = 0.0625 EU/mL.

Based the result of sensitivity test of tachypleus amebocyte lysate, the endotoxin content in the flavonoids was measured. Endotoxin standard (0.062 EU/mL) and detection water were applied as positive control and negative control. The experiments were conducted three times.

We found that the endotoxin content in flavonoids was lower than the 0.0625 EU/mL, which meet the requirement of Pharmacopoeia of the People's Republic of China.
