# Supplementary material for: Systematic analysis of bacteriostatic mechanism of flavonoids using transcriptome and its therapeutic effect on vaginitis
Source: Aging (Albany NY). 2020 Apr 9;12(7):6292–305. doi: 10.18632/aging.103024 (PMC7185132; doi:10.18632/aging.103024)
Supplement: Supplementary Tables [file aging-12-103024-s002..pdf]

Supplementary Tables

Supplementary Table 1. Sensitivity test of tachypleus amebocyte lysate.

Endotoxin (EU/mL)	0.5	0.25	0.125	0.062	0.031
1	+	+	+	+	-
2	+	+	+	+	-

Supplementary Table 2. Measurement of endotoxin content in flavonoids.

Samples	Positive control	Negative control	flavonoids
1	+	-	-
2	+	-	-
3	+	-	-
